# Correction: Dopamine modulates subcortical responses to surprising sounds

**DOI:** 10.1371/journal.pbio.3000984

**Published:** 2020-10-28

**Authors:** The PLOS Biology Staff

The Competing Interests section incorrectly states that Dr. Malmierca is an Academic Editor for PLOS Medicine. The publisher apologizes for the error. The correct statement is: Manuel S. Malmierca is an Academic Editor for PLOS Biology. The other authors have declared that no competing interests exist.
