# Supplementary material for: Dopamine modulates subcortical responses to surprising sounds
Source: PLoS Biol. 2020 Jun 19;18(6):e3000744. doi: 10.1371/journal.pbio.3000744 (PMC7329133; doi:10.1371/journal.pbio.3000744)
Supplement: Attachment — Submitted filename: Malmierca et al - PLOS BIO REV 2.pdf [file pbio.3000744.s006.pdf]

The authors have adequately addressed all of my comments. Insofar as my first point regarding histological verification, I think it's worthwhile including the histological/lesion picture as supplement material and I encourage the authors to do so. Otherwise, I am fully satisfied with all other aspects of the study. Great Job!
