# Supplementary material for: Dopamine modulates subcortical responses to surprising sounds
Source: PLoS Biol. 2020 Jun 19;18(6):e3000744. doi: 10.1371/journal.pbio.3000744 (PMC7329133; doi:10.1371/journal.pbio.3000744)
Supplement: Attachment — Submitted filename: 200328 - reviewers.docx [file pbio.3000744.s005.docx]

*Dear Dr. Roland G Roberts,*

*First of all, we would like to thank the 3 reviewers for their constructive criticisms and yourself as the editor for offering us the opportunity to resubmit a revised version of our manuscript. We are very grateful and pleased with the reviewers’ comments on our manuscript for offering insightful observations and suggestions. We have made all the necessary revisions to address all the reviewers´ comments and we think the revised version of our manuscript is sincerely much improved.*

*In the response to the reviewers below, we explain and reply in detail to the specific comments one by one as listed by the reviewers and we have highlighted in blue our responses. The changes in the manuscript are also highlighted in a separate file (Manuscript-track-changes) as suggested in your decision letter to assist you and facilitate the changes tracking.*

*We hope it is now acceptable for publication.*

*Sincerely yours,*

*Manuel S. Malmierca*

EDITOR: You'll see that all three reviewers are broadly positive about your study, but that reviewers #1 and #3 request a significant number of additional analyses, and between them the reviewers ask for several textual improvements. In particular, the Academic Editor asked me to emphasise the need to broaden the framing of the paper for those in both the dopamine and predictive coding fields, and also with our wider readership in mind. In light of the reviews (below), we will not be able to accept the current version of the manuscript, but we would welcome re-submission of a much-revised version that takes into account the reviewers' comments. We cannot make any decision about publication until we have seen the revised manuscript and your response to the reviewers' comments. Your revised manuscript is also likely to be sent for further evaluation by the reviewers.

*Dear Dr. Roland G Roberts,*

*We are very grateful and glad to tell that we have found these reviews very constructive and helpful to improve our study. We have performed all the requested analyses and expand the framing of the manuscript to make it more appealing for a wider readership. As result, the manuscript has been almost completely recast but our main message and conclusions remain unchanged. In the following, we reply point by point to each reviewer comment.*

Reviewer #1:

The Authors present the results of single-neuron recordings in the inferior colliculi of anaesthetised mice (N=31), combining auditory oddball paradigms with microiontophoretic application of dopamine or eticlopride. They observe differential effects of dopamine on responses to auditory deviants, standards, and control sounds. These effects are heterogeneous across neurons but at the population level decrease the amplitude of deviant responses. The findings are interpreted within the predictive coding framework as negative precision modulation.

Overall the manuscript is very clearly written and reports novel findings regarding dopaminergic effects on stimulus-specific adaptation. However, the interpretation of the results is less clear, given the heterogeneity of results across neurons and some methodological decisions.

*Thank you very much for your kind words and appreciation. Your review was very inspiring, and we hope that we have been able to provide now a clearer explanation on our methodology and results, as well as a more comprehensive discussion.*

Major comments:

1. Rationale: While dopaminergic gain modulation is ubiquitous in cortical and subcortical regions, its role in sensory prediction error signalling is not as obvious as implied by this manuscript. All references cited by the Authors in the introduction (line 123) and discussion (line 629) concern dopaminergic effects on reward prediction errors, not sensory prediction errors. The neural implementation of reward and sensory prediction error signalling may be quite different: for instance, reward prediction errors may be signed (positive or negative; Keller & Mrsic-Flogel, 2018), while sensory prediction errors have been suggested to be unsigned (Bastos et al., 2012). In the sensory prediction error literature, cholinergic or NMDA-ergic effects are more often reported (Moran et al., 2013; Rosch et al., 2019; Auksztulewicz et al., 2018) and dopaminergic manipulations much less common (Todd et al., 2013). Within the predictive coding framework, classical neuromodulators are often thought to increase the precision of prediction error signalling (Moran et al., 2013), contrary to the inhibitory net effects described in this manuscript; dissociations between dopaminergic and cholinergic signalling have also been proposed (Marshall et al., 2016). Taken together, the manuscript would benefit from a more comprehensive discussion of how the current results fit into the other studies on neuromodulatory effects on SSA / mismatch responses (including Authors’ own work, e.g. Ayala & Malmierca, 2015) and the broader theoretical framework.

*As required, the discussion of our manuscript has been considerably expanded. We hope it to be more comprehensive now. We have established a clear distinction between reward and sensory prediction errors (lines 500-529). We have fitted our current results into other previous studies on neuromodulation of SSA in the inferior colliculus (lines 456-467). In addition, we have attempted a better integration of our results the boarder predictive processing framework (lines 500-588). Finally, we would like to thank the reviewer for these helpful remarks, which have prompted us to include a new section to discuss the limitations of our work, featuring his/her observations (lines 554-561).*

2. Interpretation: Albeit the net effects (averaged across neurons) show reduced responses to deviants in both protocols (lines 414 and 522), the results are very heterogeneous across units. In protocol 1 (classical oddball), a similar proportion of neurons show decreased and increased CSI (39 vs. 30 respectively) following dopamine application; in protocol 2 (oddball + cascade), the numbers are not reported in text but Figure 6 suggests considerable variability across units. Nevertheless, the Authors conclude that dopamine reduces prediction error signalling, possibly due to reduced precision (as understood in the predictive coding framework). However, on grounds of the predictive coding framework, at least in the cortex, specific hypotheses have been formulated regarding (1) the laminar specificity of prediction error signalling (supragranular layers); (2) the class of neurons encoding gain modulation (pyramidal cells). It is unclear whether or how other types of neurons
(e.g. inhibitory interneurons) signal sensory prediction errors and how they might be regulated by neuromodulatory gain control. As such, the study has a potential in identifying previously unknown specific effects of dopamine on sensory prediction error signalling in the IC; however, to this end, variability across neurons should be analysed with respect to the types of cells involved (putative pyramidal cells vs. inhibitory interneurons). One possibility is that the neurons showing increased CSI might be predominantly labelled as putative pyramidal cells, while neurons showing decreased CSI might be labelled as putative inhibitory interneurons. Therefore, I would recommend performing a spike shape analysis to classify cells into these two broad categories, and testing for any differences in CSI effects between these classes.

*As recommended, this issue is also addressed in the section of ‘Limitations’ (lines 562-577). In contrast to the detailed knowledge about the cerebral cortex, it is our informed opinion that the current knowledge on the microcircuitry of the nonlemniscal IC does not allow to link concrete neuronal types to gain modulation encoding or prediction error signaling. Unlike in the cortex, the proposed spike shape analysis does not allow to classify IC neurons into putative excitatory cells and putative inhibitory interneurons. This has never been proven in the IC, and only a very recent study reports negative results on the attempt (Ito, 2020:* “Parameters related to spike waveforms (Fig. 6E), namely, spike width and peak–trough interval, were similar among cell types (Fig. 6F, G).”*).*

*Ito T. Different coding strategy of sound information between GABAergic and glutamatergic neurons in the auditory midbrain. J Physiol. 2020;598: 1039–1072. doi:10.1113/JP279296*

3.      Analysis: The Authors report baseline shifts in protocol 1 (Results, lines 427-436) in addition to effects on baseline-corrected measures. The reported baseline shifts are in line with previous proposals that precision modulation should be visible in the pre-stimulus baseline (Hesselmann et al., 2010). However, how might such robust effects of dopamine on spontaneous activity (+61% and -50% in neurons showing decreased vs. increased CSI) affect the analysis of baseline-corrected data? For instance, is the lack of a significant effect on DEV responses (p = 0.114; line 430) due to a ceiling effect given a strong increase in spontaneous firing? More generally, if I understand correctly, the baseline seems to be simply subtracted per condition from the spike-density function of each neuron (lines 298 onwards); as a result, the reported effects on evoked responses may to some extent depend on the baseline effects. Let’s consider the following scenario (based on Fig. 3E):
- The baseline-corrected spike density peak for DEV is 40 spikes/s in the control condition and 30 spikes/s following dopamine (Fig. 3E). At the same time, there is a baseline firing shift of ca. +60% (line 431)
- A neuron with very sparse spontaneous firing in the control condition (e.g. 3 spikes/s) would accordingly peak at 43 spikes/s without baseline correction; after dopamine, the increased baseline would be 4.8 spikes/s (60% more than 3 spikes/s) and the peak response at 34.8 spikes/s. Therefore, in absolute terms, this neuron would reduce its activity following dopamine
- Another neuron with very fast spontaneous firing in the control condition (e.g. 40 spikes/s) would peak at 80 spikes/s without baseline correction; after dopamine, the increased baseline would be 64 spikes/s and the peak response 94 spikes/s. Therefore, in absolute terms, this neuron would actually increase its activity following dopamine.
The Authors should consider whether and how their effects might be affected by baseline correction methods, and whether other correction methods (e.g. division or log-normalisation) wouldn’t be more desirable. For completeness, could the Authors also report the baseline effects following eticlopride administration as well as dopamine in protocol 2?

*We thank the reviewer for bringing this to our attention.*

*A relevant aspect of our preparation (recordings from the inferior colliculus of anesthetized rats) is that the spontaneous firing rates (SFR) are very low. In the previous version of the manuscript we did not report the actual SFR values (our mistake), only the % changes. Although the % changes are presumably quite large, as the reviewer notes, the actual values are of little relevance. For instance, see Fig. 2B and 3B. In control condition of the dopamine experiment the median SFR is about 1 order of magnitude smaller than the STD FR (0.18 vs 1.71 spikes/s) and the STD FR is over 30 times larger (0.18 vs 5.63). As a reference, 0.2 spikes/s means only 6 spikes happening during the 30 seconds (75 ms x 400 stimuli) considered for spontaneous activity in an oddball sequence. Furthermore, SFR changes at the population level are not significant.*

*Despite having some favorable characteristics, we found that the division and log-normalisation methods are unsuited for our data. Both methods require a division by the SFR, and a consequence of low SFR is that in some recordings SFR = 0, which has problematic consequences. Also, small absolute changes in the SFR would result in disproportionate changes in the normalized results (for example, using division normalization, changing from SFR=0.1 to 0.2 spk/s, would halve the normalized response). For these reasons, only suitable normalization would be to subtract the SFR.*

*Using SFR subtraction normalization means that evoked FR less than SFR (which could indicate inhibited responses) result in negative normalized FR. This is fine for firing rates, but the CSI calculation is not designed to work with negative values. The results become meaningless, and in some cases even fall outside of the [-1 – 1] range. We did not notice that flaw in the previous version of the manuscript. Thus, and given the small SFR found in our preparation, we have repeated the analysis but this time using the raw FR, without applying any normalization. For this reason, some of the reported values (i.e. number of units with significant changes) are different. However, the main results and conclusions are not changed after this methodological change. In addition, the real SFR values in control and drug conditions are now properly reported in the manuscript.*

Minor comments:

-       Abstract, line 33: I would suggest rephrasing as “… ‘precision’, which is theoretically encoded by the neuromodulatory (e.g., dopaminergic) systems”

*Rephrased.*

-       Introduction, lines 92-93: I would suggest rephrasing as “A neurobiologically informed theory of hierarchical perceptual inference, known as predictive coding…”

*Rephrased.*

-       Lines 105-107: While a separation of mismatch responses into the prediction error and repetition suppression components based on responses to deviants and standards (vs. controls) is advocated here by the Authors, other accounts (e.g. Garrido et al., 2010; Auksztulewicz & Friston, 2015) have suggested that the difference between deviance detection and adaptation may rather be linked to differences in mechanisms (connectivity vs. gain) or time scales (inference vs. learning)

*Rephrased here and throughout the manuscript to better adapt our interpretations to the broader predictive processing framework.*

-       Methods, line 205: which frequency range was used in the FRA recordings and subsequent experiments?

*Included: 0.7–44 kHz*

-       Line 251: Please reiterate that analyses are based on 40 trials per condition in this protocol

*Clarified.*

-       Line 295: Please clarify whether SEMs are across trials, neurons, or both

*Both, although now we express all values as “median [interquartile range]”.*

-       Line 299: What was the bin size for PSTH calculation before smoothing? How was the optimal bin size determined?

*The bin size was 1 ms. The manuscript has been amended to make it clearer. We have not used any method to determine an optimal bin size.*

-       Line 307: Were only excitatory responses considered? If so, what were the criteria for excluding inhibitory responses, and how could this influence the results? For instance, could it be the case that some neurons show an overall sensory inhibition relative to baseline in the control condition, but a release from inhibition following dopamine administration?

*Excuse us, this is a mistake, we have not proceeded as described in this study. The methods have been amended accordingly.*

-       Line 331: “a different neuronal response to DEV and CAS could only be due to the fact that DEV violates a prediction of the perceptual model whereas CAS fulfils it” – isn’t this only true under the assumption that only the immediately preceding stimulus influences the neural response? Cascades and oddball sequences are only matched with respect to one preceding stimulus.

*Rephrased.*

-       Lines 387-389: From this section it is not clear what kind of a follow-up analysis is conducted on these neurons, and whether it constitutes a form of double-dipping.

*We used the bootstrap procedure to determine which individual units experienced significant changes in their CSI due to the application of the drug. On the other hand, the Wilcoxon sign rank test was used to test for changes at the population level, both for FRs and CSI. The manuscript section describing these analyses has been rewritten, we hope it is clearer now.*

-       Lines 390-392: From this section it is not clear that the regression analysis is done across neurons and which variables are treated as predictors

*This part has been removed from the manuscript for simplicity. We believe that the iPE and iRS indices (originally included while following the methodology of Parras et al., 2017), as well as the regression analysis, were offering redundant information and blurred the main message of the study, which is that dopamine exclusively reduces DEV responses at population level.*

*Parras, G. G., Nieto-Diego, J., Carbajal, G. V., Valdés-Baizabal, C., Escera, C., & Malmierca, M. S. (2017). Neurons along the auditory pathway exhibit a hierarchical organization of prediction error. Nature Communications, 8(1), 2148. https://doi.org/10.1038/s41467-017-02038-6*

-       Results, line 419: Previously in the methods section FRA has only been described as an initial mapping, not as a repeated measurement

*Corrected.*

-       Lines 444-446: Were the effects of dopamine continuously assessed?

*We performed periodical measurements. We have rewritten ‘Methods’ to better explain this and other matters.*

-       Line 459: Was there enough statistical power, based on 8 or 15 units, to test for statistical significance?

*We acknowledge that splitting the data reduces considerably the number of available data points, which has an impact on the statistical power. The most affected would be the CSI increase and CSI decrease for the eticlopride experiment. After the reanalysis of the data, these groups contain 13 and 19 units each, respectively. In both cases, the power for detecting a large effect size (i.e. Cohen’s d = 0.8) would be quite reasonable (power = 0.70 and 0.87, respectively; calculated using G*Power, for a post-hoc Wilcoxon signed-rank test (matched pairs) with two tails, alpha = 0.05). However, the power is considerably reduced for moderate effect sizes (d = 0.5; power = 0.36 and 0.52, respectively). The actual effect size calculated for those groups is indeed rather small (d=0.28 and 0.15 for the deviant firing rate; d=0.05 and 0.07 for the standard firing rate), which is even smaller, so the design is underpowered in these cases. On the other hand, such small effects are unlikely to have a real physiological significance, that could justify a large increment in the number of recorded units.*

*This limitation is not extensive to the rest of the other groups. The rest of analogous analysis (considering all units in the eticlopride experiment, and the dopamine experiments including all units as well as the CSI increment or decrement groups) have powers >=0.78 for d = 0.5.*

*In any case, we decided that the best course of action is to avoid splitting our samples in the current version of the manuscript.*

-       Line 491: The analysis of first spike latency is not described in the methods and it is unclear why it is reported in this section but not in the other analyses

*Removed. This was part of a previous draft and should have not appeared in the manuscript, we apologize.*

-       Line 518: Please provide numbers of neurons showing increases, decreases, and stable responses

*Done.*

-       Discussion, lines 568-570: These effects were reported as not significant in the results section

*Rephrased. Eticlopride effects did not describe any significant tendencies at population level. However, 75% of our sample underwent significant CSI changes after eticlopride application.*

-       Lines 574: The statistical analysis supporting this statement was not reported in the results section

*Removed. These trials were not contemplated in the original experimental design but rather improvised when the recording stability afforded them. During a closer look, we have found some methodological pitfalls during data collection and analysis that have made us doubtful about the accuracy of these data, so we have decided to retract them.*

-       Lines 604-606 and 610: Fig. 6C suggests that many neurons also have negative mismatch index (iMM); furthermore, dopamine application brings the prediction error index (iPE) to negative values. The Authors should discuss these findings in more detail, as they go beyond the standard understanding of sensory prediction errors within the predictive coding framework.

*We have found a small percentage of neurons showing negative values of both CSI (e.g., Malmierca et al, 2008) and iMM (Parras et al, 2017) in all our previous studies in the IC, the auditory thalamus and the cortex (see Fig. 3B of Parras et al, 2017). Although comparatively unusual, some neurons consistently respond more to the STD than to the DEV. This could be related to effects of repetition enhancement that occur in absence of repetition suppression. However, we have never found a satisfactory hypothesis and data to support it. Whether the reviewer had any suggestion or insight on this matter, we would be most delighted to read and consider any possible explanation.*

*On the other hand, finding negative iPE values is rather common. The iPE is not expected to function as an index of ‘all’ prediction error, so to speak, but as an index of ‘genuine’ or ‘pure’ prediction error, as understood in mismatch negativity (MMN) literature. Whether the iPE had a positive value, this ‘genuine’ prediction error refers to the portion of the mismatch response that cannot be explained by mechanistic neural accounts of the MMN, such as the SSA in narrow frequency channels model. This model predicts stronger responses to no-repetition controls (such as CAS) than to the DEV, which would yield negative iPE values. Hence, negative iPE values are expected, while the presence of iPE values equal or larger than zero speaks in favor of a predictive processing interpretation (Taaseh et al., 2011). In any case, we have realized that addressing this discussion was the aim of Parras et al, 2017, but not of the present study. Here, iPE and iRS are only contributing to overcomplicate the main message of the paper. They would require a more detailed explanation, and even with that, those indices could be potentially misleading due to their nomenclature. Therefore, we have decided to remove everything regarding these indices from the current manuscript. Now the text focusses on comparing the DEV, STD and CAS responses directly, without adding unnecessary levels of mathematical abstraction besides the indices of SSA (CSI and iMM), which meaning is more straightforward and intuitive.*

*Parras, G. G., Nieto-Diego, J., Carbajal, G. V., Valdés-Baizabal, C., Escera, C., & Malmierca, M. S. (2017). Neurons along the auditory pathway exhibit a hierarchical organization of prediction error. Nature Communications, 8(1), 2148. https://doi.org/10.1038/s41467-017-02038-6*

*Taaseh, N., Yaron, A., & Nelken, I. (2011). Stimulus-Specific Adaptation and Deviance Detection in the Rat Auditory Cortex. PLoS ONE, 6(8), e23369. https://doi.org/10.1371/journal.pone.0023369*

-       Lines 630-636: This paragraph summarises the results in terms of negative precision modulation, focusing on the net effects (but not on heterogeneity of effects across neurons). However, it does not contextualise these findings in terms of predictions of most theoretical accounts of hierarchical perceptual inference or other neuromodulatory effects reported in the IC (see major point 1). I would suggest to expand the discussion on these topics, as it would enhance the impact of the paper as a contribution to revising the predictive coding framework.

*We agree. Accordingly, the whole section of ‘Discussion’ has been greatly expanded, trying to better contextualize our findings and interpretations in the broader predictive processing framework. We have emphasized the net effects rather than the individual effects because the causes of the latter can be manifold and impossible to track accurately. The new manuscript includes a whole subsection within ‘Discussion’ to addressing the heterogeneity of effects across neurons in detail (lines 470-496), as well as another subsection regarding the limitations of our interpretations (lines 553-577).*


-       Figures 3C and 4C: please mark statistical significance (or lack thereof) on the plots

*Done for all figures.*

-       Figure 5C: please clarify whether error bars are across the 5 neurons only

*Removed.*

Typos / grammar:

-       Line 150: “processed”
-       Line 170: “stimuli”?
-       Line 237: “- as opposed to DEV.”
-       Line 273: “tested during / in”
-       Line 273: “in a few cases”?
-       Line 331: “could only be due to the fact”
-       Line 580: “in the IC cortices, but…”
-       Line 645: “extent”

*Corrected.*

Additional references:

Auksztulewicz R, Friston K. Repetition suppression and its contextual determinants in predictive coding. Cortex. 2016;80:125–140. doi:10.1016/j.cortex.2015.11.024
Auksztulewicz R, Schwiedrzik CM, Thesen T, et al. Not All Predictions Are Equal: "What" and "When" Predictions Modulate Activity in Auditory Cortex through Different Mechanisms. J Neurosci. 2018;38(40):8680–8693. doi:10.1523/JNEUROSCI.0369-18.2018
Ayala YA, Malmierca MS. Cholinergic Modulation of Stimulus-Specific Adaptation in the Inferior Colliculus. J Neurosci. 2015;35(35):12261–12272. doi:10.1523/JNEUROSCI.0909-15.2015
Bastos AM, Usrey WM, Adams RA, Mangun GR, Fries P, Friston KJ. Canonical microcircuits for predictive coding. Neuron. 2012;76(4):695–711. doi:10.1016/j.neuron.2012.10.038
Garrido MI, Kilner JM, Kiebel SJ, Stephan KE, Baldeweg T, Friston KJ. Repetition suppression and plasticity in the human brain. Neuroimage. 2009;48(1):269–279. doi:10.1016/j.neuroimage.2009.06.034
Hesselmann G, Sadaghiani S, Friston KJ, Kleinschmidt A. Predictive coding or evidence accumulation? False inference and neuronal fluctuations. PLoS One. 2010;5(3):e9926. Published 2010 Mar 29. doi:10.1371/journal.pone.0009926
Keller GB, Mrsic-Flogel TD. Predictive Processing: A Canonical Cortical Computation. Neuron. 2018;100(2):424–435. doi:10.1016/j.neuron.2018.10.003
Marshall L, Mathys C, Ruge D, et al. Pharmacological Fingerprints of Contextual Uncertainty. PLoS Biol. 2016;14(11):e1002575. Published 2016 Nov 15. doi:10.1371/journal.pbio.1002575
Moran RJ, Campo P, Symmonds M, Stephan KE, Dolan RJ, Friston KJ. Free energy, precision and learning: the role of cholinergic neuromodulation. J Neurosci. 2013;33(19):8227–8236. doi:10.1523/JNEUROSCI.4255-12.2013
Rosch RE, Auksztulewicz R, Leung PD, Friston KJ, Baldeweg T. Selective Prefrontal Disinhibition in a Roving Auditory Oddball Paradigm Under N-Methyl-D-Aspartate Receptor Blockade. Biol Psychiatry Cogn Neurosci Neuroimaging. 2019;4(2):140–150. doi:10.1016/j.bpsc.2018.07.003
Todd J, Harms L, Schall U, Michie PT. Mismatch negativity: translating the potential. Front Psychiatry. 2013;4:171. Published 2013 Dec 18. doi:10.3389/fpsyt.2013.00171

*Thank you very much for providing us with these references. This list has been greatly useful to better frame and interpret all the comments above, as well as to guide us in providing a (hopefully) richer and more comprehensive discussion in the new version of our manuscript.*


Reviewer #2:

This manuscript describes the effect of Dopamine release on the response magnitude of unpredictable but not predictable stimulus events. The results are discussed in the framework of the propagation of prediction errors from the midbrain to higher stations and suggests a gating-role of Dopamine activity.

 A stimulus-specific adaption (SSA) paradigm was used, i.e., presenting a tone sequence containing predictable (high probability; standard) tones and, intermixed, some unpredictable (low probability; deviant) tones of a slightly different frequency than the standard. The authors show that the usual significant increase in response magnitude to deviants is reduced in the presence of dopamine in the cortex of the inferior colliculus. By contrast, the responsiveness to the standard tones is only minimally affected by the presence of dopamine. The experimental findings appear solid and the approach is based on detailed previous studies, many by the current authors, with a set of reasonable controls.

The main issue with the manuscript is the sole interpretation of the findings in a framework of predictive coding theory. While it is not unreasonable to cast the main hypotheses in such a scheme, it is not helpful to omit potential consequences or other potential interpretations since Dopamine release has been postulated to participate in a number of subcortical and cortical functions. Dopamine neurons have been shown to be activated by new stimuli or unpredicted rewards and reporting an error in the prediction of reward during learning. They also play a role in long-term potentiation, a putative cellular mechanism underlying plasticity, and in controlling membrane potential states. The proposed role of dopamine in modulating or even gating propagation of prediction error signals from the IC to higher stations seems worthwhile, yet a number of related aspects remain unclear. For example, it is not made clear how error encoding “precision” is related to changes in response magnitude to the deviant (the implicit assumption that firing follows a strike poisson rule is not necessarily valid).  Furthermore, the Dopamine is experimentally released minutes before the stimulus sequence, thus it is not related in time to a singular deviant event but is more akin to a local state-change as in a naturally slow release of Dopamine due to unknown causes. These aspects would make a more thorough discussion of the findings worthwhile for the reader.

*Thanks for pointing out these general shortcomings regarding our original approach. In the new version of the manuscript, we have made a substantial effort to discuss other potential interpretations (e.g., lines 500-515) and provide a wider view on dopaminergic function (e.g., lines 477-496). However, whether we have failed to sufficiently address the abovementioned concerns, we would like to kindly ask the reviewer to provide us with some references that could guide us to further expand and enrich our current take on the results. We would be very grateful.*

In the following are a number of questions/points that would be useful to address.

Line 95: “….explains predictable inputs away.”  To understand this phrase requires a thorough knowledge of predictive coding theory.  Of course the stimulus-based information – in addition to the expectation/predictability information – is still present.

*Rephrased. We hope that the whole ‘Introduction’ section is more comprehensive now regarding the conceptual tenets of predictive processing in general.*

Line 97: “superior … levels”.  Should be ‘proximal’ or ‘higher’

*Corrected.*

Line 116: unclear meaning of sentence: “….to adjust the relative influence of prediction error signals over perception”. Perception is not unequivocally tied to stimulus or error representation.

*Rephrased.*

Line 120: unclear meaning - reformulate: “regulates forward message-passing modulating the postsynaptic gain”

*Rephrased.*

Line 127: perhaps “…modulates response properties and possibly predictive auditory processing aspects”.

*Rephrased.*

Lone 129: “perhaps: “….IC cortices, compatible with the notion of lowering the precision of prediction signals”. But see comment above about magnitude strength and variance.

*Rephrased.*

Line 224: What are potential consequence for using 0.28 octave spacing in Protocol 1 and 0.5 oct in Protocol 2?

*In agreement with previous studies (e.g. Malmierca et al., 2009, Ulanovsky et al, 2003), we found that larger frequency separations tend to generate larger CSI values. In our sample we found that the 0.5 octave group tends to show slightly higher deviant firing rates and lower standard firing rates, and in consequence the CSI tends to be higher, but the differences between those two groups are not significant, which supports pooling them together for analysis.*

*The following table summarizes the comparison between the 0.28 and 0.5 octave groups. P-values are based in a 2-way ANOVA with Dunn-Sidak correction.*

|  | **Control** | | | **Dopamine** | | |
| --- | --- | --- | --- | --- | --- | --- |
|  | **0.28 oct** | **0.5 oct** | **p-value** | **0.28 oct** | **0.5 oct** | **p-value** |
| **DEV FR (median [IQR] spk/s)** | 5.53  [3.22-10.25] | 5.63  [3.22-9.63] | 0.994 | 4.13  [1.84-7.13] | 4.38  [1.25-7.81] | 0.887 |
| **STD FR (median [IQR] spk/s)** | 2.04  [0.88-3.56] | 1.38  [0.42-3.86] | 0.788 | 1.69  [0.42-3.82] | 1.50  [0.16-3.14] | 0.995 |
| **CSI (median [IQR])** | 0.49  [0.27-0.71] | 0.58 [0.25-0.87] | 0.544 | 0.38  [0.10-0.66] | 0.49  [0.23-0.78] | 0.227 |

Line 233: rewrite “… a bunch of tones…”; too colloquial

*Rephrased.*

Line 554: the word “gating” usually implies an all-or-nothing mechanisms.

*Replaced by “modulating” and “regulating” along the whole manuscript.*

Line 604: State the reason why you conclude this (because CAS responses were not affected….)

*Done.*

Line 615: One could add here that the differential effect of Dopamine on DEV and STD cannot be explained by differences in the initial firing rate for DEV and STD because CAS has also a relatively high firing rate and seem unaffected.

*Great suggestion, thanks. Added in lines 443-445.*

Lines 630 to 636: largely speculative and not very crisp. Especially the last line “….deeming their ‘reliability’…..” is very opaque.

*This whole section has been considerably expanded and rewritten, so we expect the text to be clearer and more comprehensive now. In addition, we have made an effort to signal and make more obvious the parts where we are indeed speculating and considering alternative interpretations (e.g., lines 530-531), so the reader does not confound the results of the present and previous studies with our own hypotheses and conjectures about the data.*


Reviewer #3:

This is a nice manuscript that looks at the role of dopamine and how it modulates stimulus specific adaption at the level of the auditory midbrain. The authors show that dopamine signaling modulates SSA in the IC cortices and that this signaling is highly correlated with and consistent with a prediction error signal (top down modulation). Below I provide comments / suggestions, particularly with details that need clarification or could be improved for correctness.

*Thank you very much for your kind words and your helpful suggestions to improve our work.*

1) The authors performed histological verification with lesions to verify that they are in cortical regions of IC. It would be highly valuable to know where exactly in the IC cortices these recordings are situated. Are the recordings in dorsal cortex, lateral cortex etc? Can the authors collapse their location information on a standardized atlas of IC? And are there differences in location between sites that exhibit significant changes with dopamine / or antagonist? I realize that a full mapping of these functional properties may be a bit beyond the scope if this initial manuscript, having slightly more detailed anatomical information as it relates to the functional properties would be valuable nonetheless.

*Previously, we had only used our histological controls to verify that our recordings were being performed in the nonlemniscal IC, and not in the central nucleus. Now, we have thoroughly reviewed our histological preparations to establish in which part of the IC cortices were the electrolytical lesions. We conclude that they are all located in the rostral cortex of the IC, which is consistent with the fact that we were not greatly varying the recording coordinates during or between experiments. We have included this information in the manuscript. We could also provide pictures of the lesions as supplementary materials as we have done in previous studies from our lab, but we feel they are somewhat redundant at this stage. Notwithstanding, whether the reviewer thinks otherwise, we would be happy to include them.*

2) Line 33 and 44 – abstract – can the authors more concisely define their usage of “precision” or alternately use a more suitable description. Its not obvious how being accurate (precise) is the relevant factor in this study. What seems to be most important is simply the fact that dopaminergic system modulates neural response in a manner the mimics prediction error signaling. This is precisely what the study shows. As far as I can tell, I don’t really see anything in the manuscript regarding precision, since the authors are not using any measures that quantify how accurate the signaling really is.

*We have rewritten the abstract to address these issues (lines 32-35). In addition, we have tried to provide more comprehensive explanations regarding the abovementioned concerns all along the manuscript (e.g., lines 112-128). The specific reason why we believe the notion of ‘precision’ is needed to interpret our results is thoroughly explained in lines 516-538.*

3) Line 196 – “spike waveforms are identical and clearly separable from other smaller units and the background noise …”

First, the waveforms may very well be very consistent and similar across spike but there is no way that they can be identical!

Have the authors quantified the waveform quality? And did you use a quantitative selection criteria other than visual inspection (not ideal) to designate single units? For example, one can compute the signal-to-noise ratio (or a multitude of other quality metrics) of the spike waveforms and can designate single units as those waveforms that meet a specific criteria (for instance SNR>5).

At minimum, it would be valuable if the authors compute a metric such as SNR for all the units and provide some statistics for the quality of their waveforms.

*We apologize for this confusing sentence. Spike waveforms from a single neuron can be very similar, but of course not ‘identical’. We have calculated the SNR for the spike waveforms for the recorded units and found that 51% had a SNR>5. We have mentioned this analysis in the Methods section and modified the manuscript accordingly, to indicate that the sample includes both single- and multi-units.*

4) Line 235 and several other locations in the MS – “in consequence” – better to use “Consequently”

*Corrected.*

5) Line 233 – “bunch of tones”, maybe better to say a “set of 10 tones” or “sequence of 10 tones”.

*Corrected.*

6) Line 266 – “microdomaine” – Is there any information the authors can provide or can you estimate the radius of these microdomains? Or even the volume of the drug delivered? It would be valuable to know the spatial extent over which the antagonist affects the IC neural circuitry.
Also, in this regard, can the authors speak towards whether the volume injection size correlates with whether a significant effect is observed or not? Im not sure this is even possible to do, but if it were it would be interesting to know.

*Although it is very relevant to know the spatial extent of effects of the applied drugs, there is little information about it. Only a handful of studies had attempted to estimate it. Uteshev et al. (2014) evaluated the diffusion of a fluorescent tracer applied by pressure and found that the concentration dropped to 50% around 40-50 µm away from the delivery site.*

*In contrast to pressure injections, microiontophoresis is a non-quantitative technique which does not allow to determine the amount of drug being ejected (Bloom, 1974; Purves, 1980). There isn’t much information either about the extent of the effects;* *Burger and Pollak (2001) report effects of kynurenic acid when the recording electrode was up to 275 µm away from the drug delivery site. For this reason, we are very careful to reproduce standard conditions for all experiments, such as using similar ejection currents, times of drug application or tip sizes, in order to reduce as much as possible the variability of the quantity of drug ejected in each case. Given that the amounts of drug applied should be equivalent, we are more inclined to think that the observed effects are rather determined by the differences in the repertoire and density of receptors expressed in each neuron.*

*Burger, R. M., & Pollak, G. D. (2001). Reversible inactivation of the dorsal nucleus of the lateral lemniscus reveals its role in the processing of multiple sound sources in the inferior colliculus of bats. J Neurosci, 21(13), 4830–4843.*

*Bloom FE (1974) To spritz or not to spritz: the doubtful value of aimless iontophoresis. Life Sci 14:1819–1834.*

*Purves R (1980) Iontophoresis — progress and pitfalls. Trends Neurosci 3:245–247.*

*Uteshev, V. V., Gusev, A. G., & Sametsky, E. A. (2014). Network Control Mechanisms-Cellular Milieu. Neuronal Networks in Brain Function, CNS Disorders, and Therapeutics. Elsevier Inc.* *https://doi.org/10.1016/B978-0-12-415804-7.00010-1*

7) Line 315 – CSI metric – If one were to compute a CSI for each sound (CSI_1, CSI_2) how does this traditional CSI compare? Is it generally correlated with both? Or does generally one or the other sound dominate?

*We chose f_1_ and* *f_2_ so their responses (based on the FRA recorded at the beginning of the procedure) were as similar as possible, to minimize the effect of different response levels. As shown in also in previous studies (e.g. Ulanovsky et al., 2003; Malmierca et al., 2009;), SI1 and SI2 (i.e., the analogous to the CSI index but calculated only for f_1_ or f_2_, respectively) tend to show relatively similar values, at least in the same direction. In this study the median difference in the distance between the CSI and either SI1 or SI2 is 0.0491 [0.0120-0.1184]. A linear fit of individual SI values vs CSI shows a high correlation (r^2^ = 0.70).*

8) Line 382 – bootstrap procedure – a bit more detail on exactly how this is done would be useful. Are you bootstrapping across trials? By requiring that the 95% confidence intervals don’t overlap, is this equivalent to a significance criteria of p<0.05?

*We have now explained the bootstrap procedure in greater detail.*

*We calculated the confidence interval for the CSI in the control condition, and then checked whether it contained the CSI value in the drug condition. According to Greenland et al. (2006), “if one of the 95 % intervals contains the point estimate from the other group or study, we will find P<0.05 for the difference”. We consider this method a good approximation, given the characteristics of the data.*

*“Greenland, S., Senn, S. J., Rothman, K. J., Carlin, J. B., Poole, C., Goodman, S. N., & Altman, D. G. (2016). Statistical tests, P values, confidence intervals, and power: a guide to misinterpretations. European Journal of Epidemiology, 31(4), 337–350. https://doi.org/10.1007/s10654-016-0149-3”*
