# Supplementary material for: Dopamine modulates subcortical responses to surprising sounds
Source: PLoS Biol. 2020 Jun 19;18(6):e3000744. doi: 10.1371/journal.pbio.3000744 (PMC7329133; doi:10.1371/journal.pbio.3000744)
Supplement: Attachment — Submitted filename: response to reviewers.docx [file pbio.3000744.s007.docx]

Dear Dr. Ronald Roberts (and also in reply to the Academic Editor),

Thanks for your support and guidance along the way. We have taken careful consideration on your feedback and improved our manuscript accordingly. We have addressed all the reviewers’ last comments, as well as the Academic Editor’s concerns.

In order to make our work more accessible and appealing to the broader readership of PLOS Biology, we now provide much more contextual information to frame the key elements of the paper and explain the comprehensive significance of our results. We have tried not to take any knowledge for granted. We have also made a big effort to change the structure of our explanations. Now, better known facts and more general aspects feature first in the text, so that the average reader of PLOS Biology can feel interested and engaged with the paper before getting into the essentials of our proposal. You will find a considerable amount of new text but note that no new ideas have been added really. Just previous ideas are explained more in depth, while preserving the original message and scientific core of the work. Finally, we have followed your advice to provide a more general title to the paper. We feel quite satisfied with these improvements to reach the full potential of the paper. Hopefully, you will feel the same way after reading this new version.

In the response to the reviewers below, we explain and reply in detail to the specific comments one by one as listed by the reviewers and we have highlighted in purple our responses. The changes in the manuscript are also highlighted to assist you and facilitate change-tracking.

Best wishes, on behalf of all authors

Manuel S. Malmierca

------------------------------------------------------------------------
REVIEWERS' COMMENTS:


Reviewer #1:

Following revisions, the manuscript has improved considerably. I only have a few minor comments:

*Thank you very much for recognizing our efforts.*

- In the discussion (lines 530-538), the Authors interpret their finding in terms of dopaminergic signalling of "imprecision" via net negative gain. This seems to be the main interpretation of their results, with major implications for predictive coding theories. However, it is unclear how such net negative gain could be signalled. Could the Authors speculate on a plausible neuroanatomical circuit mediating net negative gain, for example in terms of inhibitory and disinhibitory circuits in nonlemniscal thalamus (Chen et al., JNeurosci 2018, doi: 10.1523/JNEUROSCI.2173-17.2018)?

*Thanks for recommending Chen’s paper. This was indeed an unfortunate omission, but it has been readily incorporated along the manuscript (lines 524-527, among others). The Chen study indeed helped us to better described the neuroanatomical substrate where such precision-weighting mechanism could be implemented and provided more details about the dopaminergic innervation of the IC and where possible we have been cautious to speculate too much on more inhibitory and disinhibitory circuits for the reasons argued in the section of ‘Limitations’. Also, to make sure we have no more omissions, we asked Dr. Ito, a friend and colleague that is a coauthor in Chen’s his opinion on the inhibitory and disinhibitory IC circuits.*

*We have also provided a new figure to illustrate how these neuroanatomical structures are organized grosso modo, so the reader can find a panoramic view of the proposal.*

- In the introduction, the term "negative gain" (line 139) should be clarified

*We agree that term negative gain is unclear and confusing. We have changed it for ‘attenuation’ all along the manuscript. Attenuation is more appropriate and less confusing, thanks for drawing our attention on this pitfall.*

- I would suggest foreshadowing the discussion of sensory vs. reward prediction errors in the introduction so that the reader has a more informed impression of the literature

*Agreed. Now this discussion is covered in great length in the Introduction.*


Reviewer #3:

The authors have adequately addressed all of my comments. Insofar as my first point regarding histological verification, I think it's worthwhile including the histological/lesion picture as supplement material and I encourage the authors to do so. Otherwise, I am fully satisfied with all other aspects of the study. Great Job!

*Thank you very much for recognizing our efforts and for your enthusiastic support. Since we do not have many figures in this paper, we have decided to include a figure showing examples of these histological lesions. Hence, the broad readership which may not be used to this kind of technics can get a quick impression on what we are doing in this paper.*
